# Real-time guidance by deep learning of experienced operators to improve the standardization of echocardiographic acquisitions

**DOI:** 10.1093/ehjimp/qyad040

**Published:** 2023-11-27

**Authors:** Sigbjorn Sabo, David Pasdeloup, Hakon Neergaard Pettersen, Erik Smistad, Andreas Østvik, Sindre Hellum Olaisen, Stian Bergseng Stølen, Bjørnar Leangen Grenne, Espen Holte, Lasse Lovstakken, Havard Dalen

**Affiliations:** Department of Circulation and Medical Imaging, Norwegian University of Science and Technology, PO Box 8905, 7491 Trondheim, Norway; Clinic of Cardiology, St.Olavs University Hospital, Prinsesse Kristinas gate 3, 7030 Trondheim, Norway; Department of Circulation and Medical Imaging, Norwegian University of Science and Technology, PO Box 8905, 7491 Trondheim, Norway; Department of Circulation and Medical Imaging, Norwegian University of Science and Technology, PO Box 8905, 7491 Trondheim, Norway; Kristiansund Hospital, More and Romsdal Hospital Trust, Herman Døhlens veg 1, 6508 Kristiansund, Norway; Department of Circulation and Medical Imaging, Norwegian University of Science and Technology, PO Box 8905, 7491 Trondheim, Norway; Sintef Digital, Strindvegen 4, 7034 Trondheim, Norway; Department of Circulation and Medical Imaging, Norwegian University of Science and Technology, PO Box 8905, 7491 Trondheim, Norway; Sintef Digital, Strindvegen 4, 7034 Trondheim, Norway; Department of Circulation and Medical Imaging, Norwegian University of Science and Technology, PO Box 8905, 7491 Trondheim, Norway; Clinic of Cardiology, St.Olavs University Hospital, Prinsesse Kristinas gate 3, 7030 Trondheim, Norway; Department of Circulation and Medical Imaging, Norwegian University of Science and Technology, PO Box 8905, 7491 Trondheim, Norway; Clinic of Cardiology, St.Olavs University Hospital, Prinsesse Kristinas gate 3, 7030 Trondheim, Norway; Department of Circulation and Medical Imaging, Norwegian University of Science and Technology, PO Box 8905, 7491 Trondheim, Norway; Clinic of Cardiology, St.Olavs University Hospital, Prinsesse Kristinas gate 3, 7030 Trondheim, Norway; Department of Circulation and Medical Imaging, Norwegian University of Science and Technology, PO Box 8905, 7491 Trondheim, Norway; Department of Circulation and Medical Imaging, Norwegian University of Science and Technology, PO Box 8905, 7491 Trondheim, Norway; Clinic of Cardiology, St.Olavs University Hospital, Prinsesse Kristinas gate 3, 7030 Trondheim, Norway; Department of Internal Medicine, Levanger Hospital, Nord-Trøndelag Hospital Trust, Kirkegata 2, 7601 Levanger, Norway

**Keywords:** machine learning, artificial intelligence, real time, operator feedback, echocardiography

## Abstract

**Aims:**

Impaired standardization of echocardiograms may increase inter-operator variability. This study aimed to determine whether the real-time guidance of experienced sonographers by deep learning (DL) could improve the standardization of apical recordings.

**Methods and results:**

Patients (*n* = 88) in sinus rhythm referred for echocardiography were included. All participants underwent three examinations, whereof two were performed by sonographers and the third by cardiologists. In the first study period (Period 1), the sonographers were instructed to provide echocardiograms for the analyses of the left ventricular function. Subsequently, after brief training, the DL guidance was used in Period 2 by the sonographer performing the second examination. View standardization was quantified retrospectively by a human expert as the primary endpoint and the DL algorithm as the secondary endpoint. All recordings were scored in rotation and tilt both separately and combined and were categorized as standardized or non-standardized. Sonographers using DL guidance had more standardized acquisitions for the combination of rotation and tilt than sonographers without guidance in both periods (all *P* ≤ 0.05) when evaluated by the human expert and DL [except for the apical two-chamber (A2C) view by DL evaluation]. When rotation and tilt were analysed individually, A2C and apical long-axis rotation and A2C tilt were significantly improved, and the others were numerically improved when evaluated by the echocardiography expert. Furthermore, all, except for A2C rotation, were significantly improved when evaluated by DL (*P* < 0.01).

**Conclusion:**

Real-time guidance by DL improved the standardization of echocardiographic acquisitions by experienced sonographers. Future studies should evaluate the impact with respect to variability of measurements and when used by less-experienced operators.

**ClinicalTrials.gov Identifier:**

NCT04580095

## Graphical Abstract

**Graphical Abstract:**
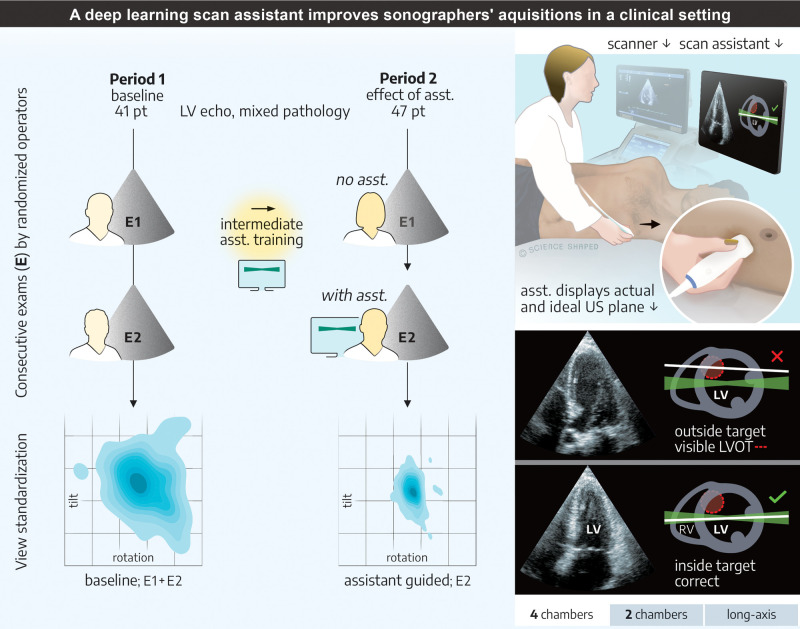
Study design, core results, and the deep learning (DL) scan assistant. Left: the characteristics of the two study periods and the repeated examinations of each patient by different sonographers. Plots depict the spread of view standardization of apical views in rotation and tilt, as evaluated retrospectively by the DL algorithm. Right: setup during patient examination and the scan assistant’s feedback to cases of incorrect vs. correct apical four-chamber view acquisitions. US, ultrasound; LV, left ventricle; RV, right ventricle.

## Introduction

Echocardiography provides essential information about cardiac morphology and function, making it a central tool for clinical decision-making in cardiology.^1^ However, echocardiographic measurements have considerable variability, even in the hands of experienced operators.^2,3^ This reduces the sensitivity to detect subtle changes in cardiac function. Impaired view standardization is an important source of measurement variability, as standardized views form the basis of obtaining reliable analyses. It has been shown that the variability introduced by the recordings is equal to that of the analyses for most left ventricular (LV) function measurements.^4–6^ Thus, expert consensus emphasizes the importance of reporting image quality and improving the standardization of recordings for optimal diagnostics and treatment in cardiology.^1,7^ However, the evaluation of view standardization has been subjective and rarely quantified in the clinic and research.

Novel ultrasound imaging analysis by deep learning (DL) can process echocardiographic images in real time.^8,9^ Beyond the measurements of cardiac function, such methods have shown the potential to improve the view standardization of echocardiographic recordings during scanning.^10,11^ Thus, we aimed to study whether the use of a DL scan assistant to guide experienced sonographers in real time could optimize the three apical views to better comply with the current recommendations. This was first evaluated using a retrospective assessment of view standardization by a human expert. Secondly, the effect of the scan assistant was evaluated using retrospective DL standardization assessment.

## Methods

### Study population

**Figure 1:**
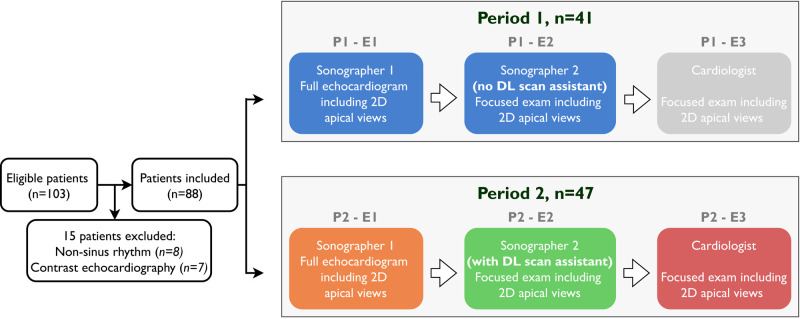
Flowchart of the study population. The same colour codings for operator groups are also used in *Figures 3–5*. E, examination; P, period.

A total of 88 participants with mixed cardiac pathology were prospectively included from the St. Olavs University Hospital’s echocardiography laboratory (*Figure 1*). Inclusion criteria were (i) indication for comprehensive echocardiography and (ii) ability to provide written informed consent. Patients with non-sinus rhythm or indication for contrast echocardiography were excluded. Inclusion was restricted to pre-defined dates based on the availability of location and personnel. The study was approved by the regional ethical committee (Central Norway 2019/7160) and performed according to the Helsinki Declaration.

### Study design

All participants underwent three consecutive examinations. The participants stayed in a supine position between the examinations, and the time delay between examinations was minimized to the change of operators only. The first and second examinations were performed by two (of three) sonographers, and the third examination was performed by one (of four) expert in echocardiography (all cardiologists). All operators were highly experienced. The sonographers all had more than 5 years of echocardiographic experience and had performed a minimum of >2000 comprehensive echocardiograms before the study started.

The data collection was divided into two separate periods (*Figure 1*). In the first period (Period 1), the sonographers were instructed to provide optimal echocardiograms for the comparative analyses of LV function. However, they were not explicitly informed about the aim of the study, the content of the training period, or the second data collection period. After Period 1 of data collection, the sonographers were introduced to the real-time DL scan assistant and trained on 10 patients each. In the second period (Period 2), the sonographer who performed the first examination (Sonographer 1) did this similarly as in Period 1, except for now being aware of the study aims and recently being trained in the use of the real-time DL scan assistant. The sonographer who performed the second examination (Sonographer 2) used the real-time DL scan assistant during scanning. The three sonographers participating in the study were randomly allocated to the role of Sonographer 1 or 2, ensuring that the sonographer examination with guidance was not performed by the same individual in all inclusions. This design was chosen to account for inter-operator differences in the standardization of acquisitions.

### Echocardiographic image acquisition

Examinations were performed with the patient in a left lateral decubitus position using a Vivid E95 scanner (version 202, GE Vingmed Ultrasound, Horten, Norway) with a phased array transducer (4Vc). For each recording, three consecutive cardiac cycles were obtained. The first examination was a comprehensive echocardiogram according to ASE/EACVI guidelines.^1^ The second and third exams were focused on the study aims and included among others 2D B-mode (greyscale) apical two-chamber (A2C), apical four-chamber (A4C), and apical long-axis (ALAX) recordings for the analyses of LV size and function. All images were acquired using second harmonic imaging (1.7/3.4 MHz), and LV-focused images were obtained using depth settings of 12–16 cm with a sector width of 65–75°. Gain settings and time gain compensation were also adjusted by the operator. All study-specific recordings were blinded and de-identified before being stored on the hospital’s imaging server.

### Real-time DL scan assistant

**Figure 2:**
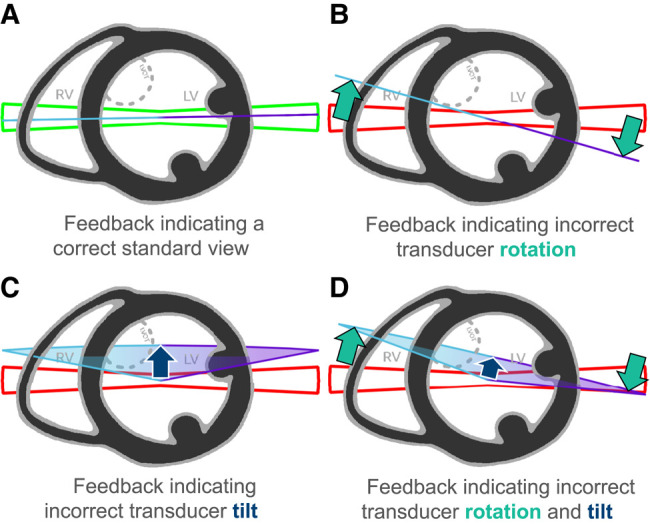
Rotation and tilt df in correct and incorrect A4C imaging planes. The imaging plane orientation is illustrated on a mid-ventricular parasternal short-axis cross section. LVOT, left ventricular outflow tract; RV, right ventricle.

The technical aspects of the real-time DL scan assistant used in this study have previously been described in detail.^10^ It has two main components: a core DL algorithm automatically analysing the images and a software application with a graphical user interface running the DL algorithm in real time while interacting with the operator (*Graphical Abstract*). The core DL algorithm estimates the position of the 2D imaging plane relative to the heart for the rotation and tilt degrees of freedom (dfs), where rotation indicates the rotational movement of the transducer, e.g. from A4C towards A2C or ALAX. Similarly, tilt indicates the tilting movement of the imaging plane away from the centreline, like anterior or posterior movement in A4C and lateral or septal movement in A2C (see Supplementary data online, *Figure S1*). Both tilt and rotation are exemplified in *Figure 2*. Unlike traditional classification of DL methods, which are only trained on standard views,^8,12,13^ this method is also trained using non-standard views to enhance spatial understanding. This approach allows to estimate the deviation from the target standard view and suggests transducer tilt and rotation movements to achieve the respective standard view. The core DL algorithm is implemented in the FAST framework,^14^ to form a complete real-time application that receives ultrasound images from a commercial ultrasound scanner and generates intuitive feedback on how to move the transducer to obtain the standardized view. A screen capture of the application is shown in the *Graphical Abstract*, and Supplementary data online, *Video S1* shows an example of the method used in real time, with both standardized and non-standardized apical views.

### Retrospective view standardization evaluation by a human expert

**Figure 3:**
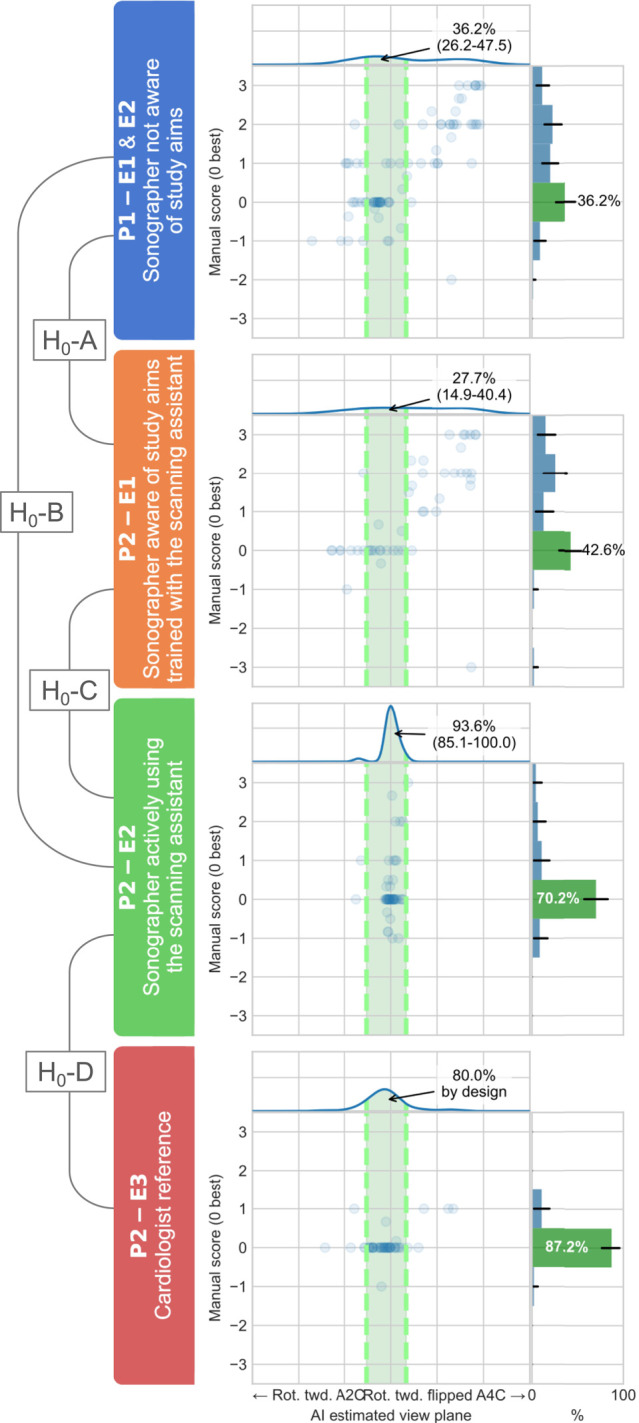
Visualization of the standardization assessment according to examinations, study periods, and hypotheses. Here exemplified by the rotational transducer position of ALAX view recordings. The distribution of DL algorithm scores is shown along the *x*-axis, while the distribution of the expert’s manual scores is shown along the *y*-axis. The colour-coded left panel indicates operators, periods, whether the DL guidance algorithm was used or not, and relation to the four null hypotheses. The reference interval from the cardiologists’ distribution is shown by the dotted line interval. E, examination; H_0_A-D, null hypotheses tested; P, period.

One echocardiography expert cardiologist (H.D.) manually evaluated view standardization in all recordings blinded to the study period, operator details, and whether the scan assistant was used or not. Rotation and tilt deviations were evaluated individually on a continuous scale from −3 to 3 in all three cycles for A2C, A4C, and ALAX recordings (*Figure 3*). A score of 0 indicated a perfect view, and negative or positive values reflected the direction and amount of deviations from the optimal alignment of rotation and tilt (see Supplementary data online, *Table S1*). Correct view standardization was arbitrarily pre-defined as a score between −0.5 and 0.5. The human expert who performed a retrospective evaluation was not involved in the development of the scan assistant.

### Retrospective view standardization evaluation by DL

All recordings were retrospectively analysed by the DL algorithm, which assigned them a relative tilt and rotational position on a continuous scale. We defined correctly the standardized recordings for the DL standardization evaluation to be within the centre 80% along the relative positional axis of the cardiologists’ recordings (*Figure 3*). The 80% position interval was arbitrarily pre-defined.

### Statistics

Following the study’s aims, four null hypotheses were tested:

1. H_0_-A: The proportion of standardized recordings is the same for sonographers before and after learning to use the scan assistant.
2. H_0_-B: The proportion of standardized recordings is the same for sonographers who are actively using the scan assistant and those who have never used the scan assistant.
3. H_0_-C: The proportion of standardized recordings is the same for sonographers who are actively using the scan assistant and those with training in, but not actively using the scan assistant.
4. H_0_-D: The proportion of standardized recordings is the same for sonographers who are actively using the scan assistant and for cardiologists not using the scan assistant.

We investigated these null hypotheses according to the primary and secondary endpoints. The primary endpoint for Hypotheses A–D was improvement in view standardization as evaluated in the human expert image analysis, and the secondary endpoint was based on the DL image analysis. All hypotheses were tested in the rotational df, tilt df, and combination of rotation and tilt dfs. The data obtained by the cardiologists in Period 1 were not included in these analyses. Statistical testing for differences in proportions between groups was calculated using the *χ*^2^ tests. Bootstrapping (10 000 resamples) was used to estimate 95% confidence intervals (CIs) for the proportions of standardized recordings. All analyses were performed with Python 3.9 using Scipy 1.10.10. *P*-values <0.05 were considered statistically significant. Using SamplePower (version 3; IBM Statistics, NY, USA), the inclusion of 38 individuals in each group provided 80% power to detect an increase in the proportion of standardized recordings from 50 to 80% (*α* = 0.05) by use of the scan assistant.

## Results

### Study population

Of 103 recruited participants, those not in sinus rhythm (*n* = 8) or with an indication for contrast echocardiography (*n* = 7) were excluded. Thus, a total of 88 patients (54% women) were included. The baseline characteristics of the study population and the key echocardiographic measurements are shown in *Table 1*. In short, the mean [standard deviation (SD)] age was 61 (17) years, 34 (38%) had heart failure or previous myocardial infarction, while 16 (18%) had moderate or severe valvular disease. Only three (3%) had chronic obstructive pulmonary disease. Sonographers using the scan assistant used more time to obtain the three apical views, with a mean of 175.7 s per examination compared with 61.7 s in sonographers without assistance (*P* < 0.001).

**Table 1:** Basic characteristics of the study population according to study periods

	Period 1	Period 2
Included study participants, *n*	41	47
Age	62 (18)	61 (15)
Women, *n* (%)	19 (45%)	29 (38%)
Clinical characteristics		
Heart failure, *n* (%)	8 (20%)	8 (17%)
Acute myocardial infarction, *n* (%)	10 (24%)	8 (17%)
Moderate or severe valvular disease, *n* (%)	7 (17%)	9 (19%)
Chronic obstructive pulmonary disease, *n* (%)	2 (5%)	1 (2%)
Body mass index, kg/m^2^	26 (4)	26 (3)
Heart rate, bpm	66 (13)	69 (10)
Systolic blood pressure, mmHg	143 (25)	136 (23)
Echocardiographic characteristics		
LV ejection fraction, %	54 (13)	55 (11)
LV end-diastolic volume, biplane, mL	124 (53)	120 (49)

Values are presented as mean (SD) or numbers (proportions).

### Primary endpoint: impact on view standardization evaluated by a human expert

**Figure 4:**
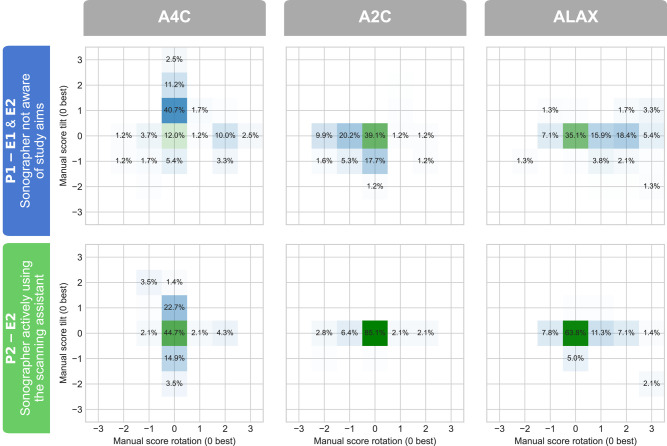
Blinded view standardization evaluation by an echocardiography expert, with rotation presented along the *x*-axis and tilt along the *y*-axis. The percentages inside the boxes indicate the proportions of recordings with a given score. E, examination; P, period.

The proportions of standardized recordings as evaluated by the human expert are presented in *Table 2* and visualized in *Figure 4*. For rotation and tilt combined, sonographers using the scan assistant had significantly more standardized recordings than sonographers without assistance in Period 1 for all three apical views (*P* < 0.01). Similar results were also found for individual dfs for A2C tilt, A2C rotation, and ALAX rotation (*P* < 0.01) and also for A4C rotation and A4C tilt (*P* = 0.069 and *P* = 0.065, respectively). ALAX tilt was numerically improved, but the difference was not statistically significant (*P* = 0.192).

**Table 2:** Proportion of standardized acquisitions according to human expert evaluation

	Period 1	Period 2			*P*-values according to hypotheses			
	1: All Son.	2: Son. without DL	3: Son. with DL	4: Cardiologist	1 vs. 2 (A)	1 vs. 3 (B)	2 vs. 3 (C)	3 vs. 4 (D)
Rotation								
A4C	71.6 (61.7, 81.5)	74.5 (61.7, 87.2)	87.2 (76.6, 95.7)	83.0 (72.3, 93.6)	0.885	0.069^a^	0.190^a^	0.772^a,b^
A2C	56.8 (45.7, 67.9)	85.1 (74.5, 93.6)	85.1 (74.5, 93.6)	83.0 (72.3, 93.6)	0.002	0.002^a^	1.000	1.000^a,b^
ALAX	36.2 (26.2, 46.2)	42.6 (27.7, 57.4)	70.2 (57.4, 83.0)	87.2 (76.6, 95.7)	0.606	<0.001^a^	0.013^a^	0.078^b^
Tilt								
A4C	30.9 (21.0, 40.7)	40.4 (25.5, 55.3)	48.9 (34.0, 63.8)	76.6 (63.8, 87.2)	0.366	0.065^a^	0.534^a^	0.010
A2C	71.6 (61.7, 81.5)	68.1 (53.2, 80.9)	100.0 (100.0, 100.0)	85.1 (74.5, 93.6)	0.826	<0.001^a^	<0.001^a^	0.018^a,b^
ALAX	81.2 (72.5, 88.8)	91.5 (83.0, 97.9)	91.5 (83.0, 97.9)	97.9 (93.6, 100.0)	0.192	0.192^a^	1.000	0.358^b^
Rotation and tilt								
A4C	12.0 (7.9, 16.2)	23.4 (16.3, 30.5)	44.7 (36.9, 53.2)	59.0 (50.4, 67.6)	0.153	<0.001^a^	0.050^a^	0.239^b^
A2C	39.1 (32.9, 45.3)	61.0 (53.2, 68.8)	85.1 (79.4, 90.8)	71.6 (63.8, 78.7)	0.027	<0.001^a^	0.016^a^	0.182^a,b^
ALAX	35.1 (29.3, 41.0)	40.4 (32.6, 48.9)	63.8 (56.0, 71.6)	85.8 (80.1, 91.5)	0.686	0.003^a^	0.039^a^	0.027

A, H_0_-A; B, H_0_-B; C, H_0_-C; D, H_0_-D; Son., sonographer.

Data shown as proportion (95% CI) % of correctly standardized views.

^a^Proportion of standardized views numerically higher when using the scanning assistant (significant and non-significant differences).

^b^Sonographer recording not significantly less standardized compared with cardiologists.

Compared with cardiologists, sonographers using the scan assistant were not significantly less standardized in A4C and A2C views for combined rotation and tilt (*P* = 0.239 and *P* = 0.182, respectively). In ALAX combined rotation and tilt, cardiologists were significantly more standardized than sonographers using the scan assistant (*P* < 0.05).

### Secondary endpoint: impact on view standardization evaluated by DL

**Figure 5:**
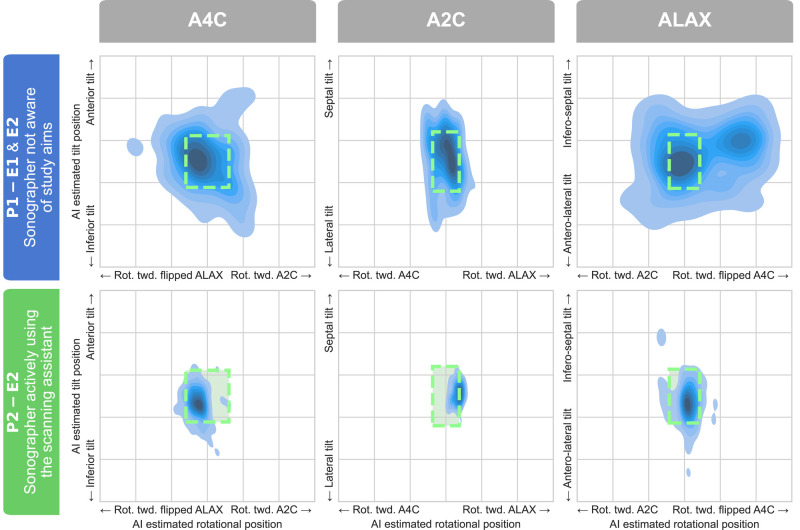
Retrospective automatic view standardization as estimated by the DL algorithm. The dotted line boxes indicate the standardization reference, as defined by the centre 80% of view orientation in the cardiologist recordings. E1, examination 1; E2, examination 2; P1, period 1; P2, period 2; Rot. twd., rotation towards.

**Figure 6:**
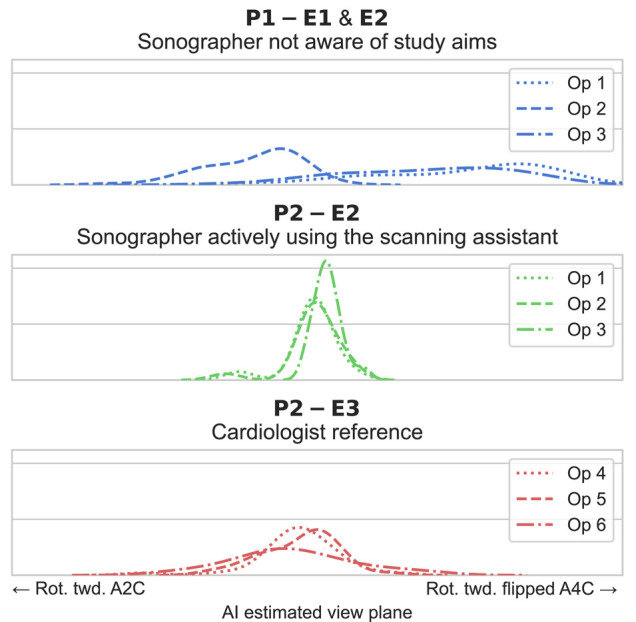
Operator-specific distributions for relative view standardization with respect to transducer rotation in the ALAX view. E, examination; Op, operator; P, study period; Rot. twd, rotation towards.

The proportions of recordings with correct standardization as evaluated by the core DL algorithm are presented in *Table 3*. *Figure 5* provides a qualitative visualization of the distribution of the estimated position along each df. For rotation and tilt combined, sonographers using the scan assistant in Period 2 significantly improved the standardization of A4C and ALAX acquisitions compared with sonographers in Period 1 (*P* < 0.001). For A2C combined rotation and tilt, the difference was not statistically significant (*P* = 0.936). However, A2C tilt was significantly improved (*P* < 0.001), while A2C rotation had a significantly lower proportion of correct recordings (*P* = 0.002). For other views and dfs individually, sonographers using the scan assistant significantly improved the proportions of correct acquisitions compared with sonographers in Period 1 in A4C and ALAX rotation (*P* < 0.001) and all three views for tilt (*P* < 0.05). *Figure 6* illustrates the operator-specific distributions for the rotational df in ALAX recordings. Similar distributions for the other apical views and dfs are included in Supplementary data online, *Figures S2–S6*. Compared with cardiologists, sonographers using the scan assistant were not significantly less standardized in combined rotation and tilt for all the three apical standard views (*P* ≥ 0.099).

**Table 3:** Proportion of standardized acquisitions according to the core DL algorithm analysis

	Period 1	Period 2			*P*-values according to hypotheses			
	1: All Son.	2: Son. without DL	3: Son. with DL	4: Cardiologist	1 vs. 2 (A)	1 vs. 3 (B)	2 vs. 3 (C)	3 vs. 4 (D)
Rotation								
A4C	66.7 (56.8, 76.5)	70.2 (57.4, 83.0)	97.9 (93.6, 100)	80.0 by design	0.827	<0.001^a^	<0.001^a^	0.116^a,b^
A2C	80.2 (71.6, 88.9)	72.3 (59.6, 85.1)	53.2 (38.3, 68.1)	80.0 by design	0.417	0.002	0.088	0.008
ALAX	36.2 (26.2, 47.5)	27.7 (14.9, 40.4)	93.6 (85.1, 100)	80.0 by design	0.425	<0.001^a^	<0.001^a^	0.073^a,b^
Tilt								
A4C	81.5 (72.8, 90.1)	83.0 (72.3, 93.6)	95.7 (89.4, 100)	80.0 by design	1.000	0.043^a^	0.094^a^	0.094^a,b^
A2C	72.8 (63.0, 82.7)	78.7 (66.0, 89.4)	100.0 (100, 100)	80.0 by design	0.597	<0.001^a^	0.003^a^	0.001^a,b^
ALAX	71.2 (61.3, 81.2)	66.0 (53.2, 78.7)	93.6 (85.1, 100)	80.0 by design	0.671	0.005^a^	0.002^a^	0.199^a,b^
Rotation and tilt								
A4C	49.8 (43.6, 56.0)	50.4 (42.6, 58.9)	85.8 (80.1, 91.5)	80.0 by design	1.000	<0.001^a^	<0.001^a^	0.217^a,b^
A2C	54.3 (48.1, 60.9)	47.5 (39.0, 56.0)	56.7 (48.9, 64.5)	80.0 by design	0.576	0.936^a^	0.491^a^	0.944^b^
ALAX	30.5 (24.7, 36.4)	19.9 (13.5, 26.2)	85.8 (80.1, 91.5)	80.0 by design	0.268	<0.001^a^	<0.001^a^	0.099^a,b^

A, H_0_-A; B, H_0_-B; C, H_0_-C; D, H_0_-D; Son., sonographer.

Data shown as proportion (95% CI) % of correctly standardized views.

^a^Proportion of standardized views numerical higher when using the scanning assistant (significant and non-significant differences).

^b^Sonographer recordings not significantly less standardized compared with cardiologists.

## Discussion

This study investigated the effect of a real-time scan assistant in optimizing the standardization of apical views by experienced sonographers. The effect was quantitatively evaluated by using retrospective human expert analysis and DL analysis. The main finding was that experienced sonographers using the scan assistant significantly improved the proportion of standardized apical recordings.

### Comparison with prior studies of real-time guidance

To the best of our knowledge, this is the first study to quantify the standardization of recordings by experienced echocardiographers. Furthermore, it is the first study to show the distribution of view standardization and the potential benefits of real-time guidance to optimize the standardization of apical views. Even though comparative studies are lacking, previous studies from our centre and elsewhere do not show the poorer standardization of recordings at our centre,^5,15,16^ indicating a potential to improve the quality in echocardiographic laboratories across the world.

Some studies have presented DL-based software for the real-time guidance of operators. Narang *et al*.^17^ and Schneider *et al*.^18^ assessed the guidance of inexperienced operators (nurses or medical students without any prior echocardiographic experience). In comparison, the experienced sonographers in the presented study had more than 5 years of echocardiographic experience with a minimum of >2000 comprehensive echocardiograms performed each. In the studies by Narang *et al*.^17^ and Schneider *et al*.^18^, machine learning–based metrics were used to evaluate image quality and displayed as a bar on the screen. In the presented study, the real-time scan assistant guided the operator by visualizing the view plane in real time and intuitively suggested adjustments of the transducer position to obtain the standardized imaging plane. Narang *et al*.^17^ and Schneider *et al*.^18^ also evaluated image quality retrospectively concerning its applicability for the qualitative estimation of cardiac volumes and systolic parameters, whereas the retrospective human scoring in the presented study evaluated view standardization based on pre-defined parameters related to the presence of specific heart structures in the images. Thus, the previously published studies differed from the presented by evaluating more liberal endpoints in less-experienced operators. Furthermore, Narang *et al*.^17^ and Schneider *et al*.^18^ did not randomize the operators for the use of guidance but used an experienced sonographer without guidance as reference. Thus, these two studies did not fully evaluate the isolated effect of guidance itself, as performed in the presented study.

### Improving view standardization by real-time guidance

For experienced sonographers using the scan assistant, the proportion of standardized recordings as evaluated by the blinded human expert (primary endpoint) was significantly improved for the combination of tilt and rotation in all three apical views compared with sonographers not using the assistant. Thus, the proposed real-time scan assistant contributed to better standardization. This finding adds to previous studies by showing the effect of the guidance itself and by proving that it is possible to improve view standardization even among experienced operators. Importantly, this also strengthens the possibility of reducing test–retest variation in clinical echocardiography performed across high-standard echocardiographic laboratories. In line with the results from manual expert analyses, the retrospective DL analyses (secondary endpoint) showed that sonographers using the scan assistant had significantly higher proportions of well-standardized acquisitions than those without the assistant in all views and dfs, except for A2C rotation and A2C combined rotation and tilt. The finding of similar results using the DL analysis and the human expert as a reference indicates the robustness of the DL method to assess image standardization. Although we did not test the within-patient reliability of the DL method, the data shown in *Figure 6* and Supplementary data online, *Figures S2–S6* indicate highly reliable and repeatable outcomes across operators. Furthermore, it concludes that the results were not provided by methodological concerns as the automatic retrospective DL image analysis used the same core algorithm as the real-time DL guidance scan assistant. As shown by the operator-specific distributions presented in *Figure 6* and Supplementary data online, *Figures S2–S6*, the individual view-specific preferences in echocardiographic imaging planes were distinct in Period 1 where the scan assistant was not used. In contrast, the distributions were narrower and more consistent across the operators in Period 2 where the scan assistant was used. This effect was most pronounced for the rotational position of the transducer in the ALAX view.

For the A2C view, the DL standardization analysis revealed a reduced proportion of recordings with correct rotational alignment for sonographers using the scan assistant compared with sonographers in Period 1 and cardiologists. At first glimpse, this could indicate that the scan assistant had a negative effect on standardization in the A2C view. However, as shown by the distributions across and within operator groups, the sonographers using the scan assistant were the most consistent in the rotational df. The choice of reference method used in this study, based on the central 80% of the cardiologists’ distribution, largely explains the reduced proportion of guided A2C images classified as standardized. The cardiologists’ A2C recordings were rotated more towards A4C compared with what was proposed by the DL algorithm as a correctly standardized A2C (*Figure 5* and Supplementary data online, *Video S2*). This finding indicates that optimizing alignment is equally important among experienced cardiologists and that the sonographers using the scan assistant obtained more standardized recordings than the reference by cardiologists. Even though we did not collect data on the perceived usability by the individual operators, the sonographers stated that the scan assistant was easy to use and provided intuitive and consistent feedback.

### Clinical implications

Slight variations in angulation and position of the ultrasound transducer may influence the recorded view and thus cause variations in measurements from repeated recordings.^3^ The recommended strategy has been to use the same operator for patient follow-up to reduce test–retest variability. However, ensuring the same operator during follow-up is logistically challenging in hospitals, and intra-operator variability is also significant.^19^ Thus, ensuring optimal standardization of acquisitions is of the highest interest for echocardiographic laboratories worldwide and may improve test–retest variability of measurements and in-hospital workflow. The presented study shows that the proposed scan assistant has the potential to improve the standardization of echocardiographic recordings across experienced users.

Diagnostic ultrasound by medical residents with basic echocardiography training has been shown to improve in-hospital diagnostic quality,^20^ and improving the standardization of acquisitions by inexperienced operators may broaden the clinical benefits of echocardiography. Currently, the learning structure in ultrasound training is based on feedback from human mentors, who may have different subjective preferences. The consistent feedback like the one provided by the DL scan assistant could improve acquisition habits and aid in echocardiographic training. The impact of such training should be evaluated in future studies.

Large echocardiographic databases are commonly used for research purposes and the training of novel DL tools.^13,21^ The quality of such datasets has usually been evaluated without objective quantitative evaluation and assumed to be of sufficient quality by citing the source or addressing the experience of the operators. Automatic standardization assessment could provide a quantitative and objective insight into the quality of echocardiographic databases, and the consistent results between the human expert’s and the DL algorithm’s view standardization analyses in this study show the robustness of the DL algorithm for this purpose.^13,21^ In a recent study, our group also showed the high validity of fully automatic quantification of LV foreshortening,^22^ another key parameter indicating the standardization of echocardiographic recordings. Several vendors and developers have created methods that automatically recognize the different echocardiographic views. Together, such methods form the basis for a standardized evaluation of the quality of echocardiographic databases used for research purposes. Hopefully, this may aid in interpretation across studies in the future.

### Strengths and limitations

The study has some noticeable strengths. First, the operators in our study were experienced sonographers and cardiologists working in an echocardiography laboratory accredited by the European Association of Cardiovascular Imaging. As most echocardiograms are performed in well-qualified echocardiography laboratories, this adds to the generalizability of the study results. Secondly, the use of two different endpoints to evaluate view standardization and similar findings across these endpoints strengthens the results.

This study also has some limitations. First, the DL scan assistant assumes the correct positioning of the transducer at the apex, indicating no LV foreshortening. LV foreshortening is a common problem in echocardiography,^23^ but a recent study from our research group showed that experienced sonographers had little foreshortening (2–5 mm).^22^ Nevertheless, future development of the DL scan assistant should also include foreshortening guidance to further optimize the anatomical orientation of apical views. Secondly, the study sample was modest, and including a larger population constituting the full spectrum of normal and diseased hearts could have made minor differences between operator groups more evident. Only patients with non-sinus rhythm or need for contrast echocardiography were excluded, and inclusion was planned to restricted dates based on the availability of operators to both serve the clinical needs and align with the study methodology. Thus, despite the modest sample size, we believe that the results are generalizable to populations commonly found in echocardiography laboratories.

The nature of the real-time scan assistant implies that learning to use the software increases attention to view standardization, which could have biased the results. To anticipate this issue, we performed inclusions in Period 1 before the sonographers were informed about the study’s aims and trained in using the scan assistant. The choice of training period and number of training subjects were pre-defined to provide sufficient training in the use of the DL scan assistant, and a different training scheme could affect the results. However, our results show only a minimal training effect during the study, and the most standardized recordings were those acquired by using the DL scan assistant. Furthermore, sonographers were randomized at each patient inclusion to use the scan assistant in Period 2. This enabled us to differentiate the learning effect, the direct guidance effect, and the combined learning and guidance effect. However, accounting for the learning effect resulted in different patients being included across the two periods. Furthermore, a slight variation in the proportion of recordings for each sonographer between periods constitutes a limitation (Supplementary data online, *Figure* *S7*). Nevertheless, analyses within Period 2 (i.e. learning effect vs. direct guidance effect) showed significant improvements in standardization in sonographers with the scan assistant, indicating a positive effect of real-time guidance on top of the learning effect.

Furthermore, sonographers who used guidance used ∼2 min more to obtain the three apical views. This constitutes a potential limitation for clinical implementation, but the sonographers were trained on just 10 patients each and the study design required guidance by an additional screen. Thus, we expect that the time used will be reduced with long-term use and implementation of the DL method into the scanner. Lastly, the thresholds of −0.5 to 0.5 for manual and 80% interval for DL analysis were arbitrarily pre-defined before analysing the results, and other thresholds could have influenced the results.

### Future perspectives

Future studies should evaluate the clinical impact of real-time guidance of operators at different levels of experience and patient populations in multicentre studies. Additionally, future studies should assess the effect of guidance combined with manual or automatic measurements to reduce the test–retest variability of central echocardiographic measurements. With the refinement of DL methods to be able to both guide operators and quantify view standardization of stored data, there is a need for a broad consensus on correct view standardization and how to report such findings. Ultimately, the guidance of experienced operators in high-quality echocardiography laboratories may improve standardization and the sensitivity to detect subtle changes in cardiac anatomy and function.

## Conclusion

Sonographers using the proposed real-time DL scan assistant to optimize the recordings of the three standard apical views had significantly higher proportions of standardized acquisitions than those not using the scan assistant. Our findings show that real-time guidance by DL can improve the standardization of echocardiographic acquisitions when used by experienced personnel in high-quality echocardiographic laboratories. The impact on the test–retest variability of measurements, the impact on the performance of the scan assistant in less-experienced operators, and the clinical impact must be addressed in future studies.

## Supplementary Material

qyad040_Supplementary_Data

## Data Availability

The dataset can be made available from the corresponding author upon reasonable request.
