# Supplementary material for: Real-time guidance by deep learning of experienced operators to improve the standardization of echocardiographic acquisitions
Source: Eur Heart J Imaging Methods Pract. 2023 Nov 27;1(2):qyad040. doi: 10.1093/ehjimp/qyad040 (PMC11195719; doi:10.1093/ehjimp/qyad040)
Supplement: qyad040_Supplementary_Data [file qyad040_Supplementary_Data.zip › Supplementary_sigbjorn_eurheart.docx]

**Supplemental Figure 1.** Degree of rotation and tilt for the human expert scoring system.


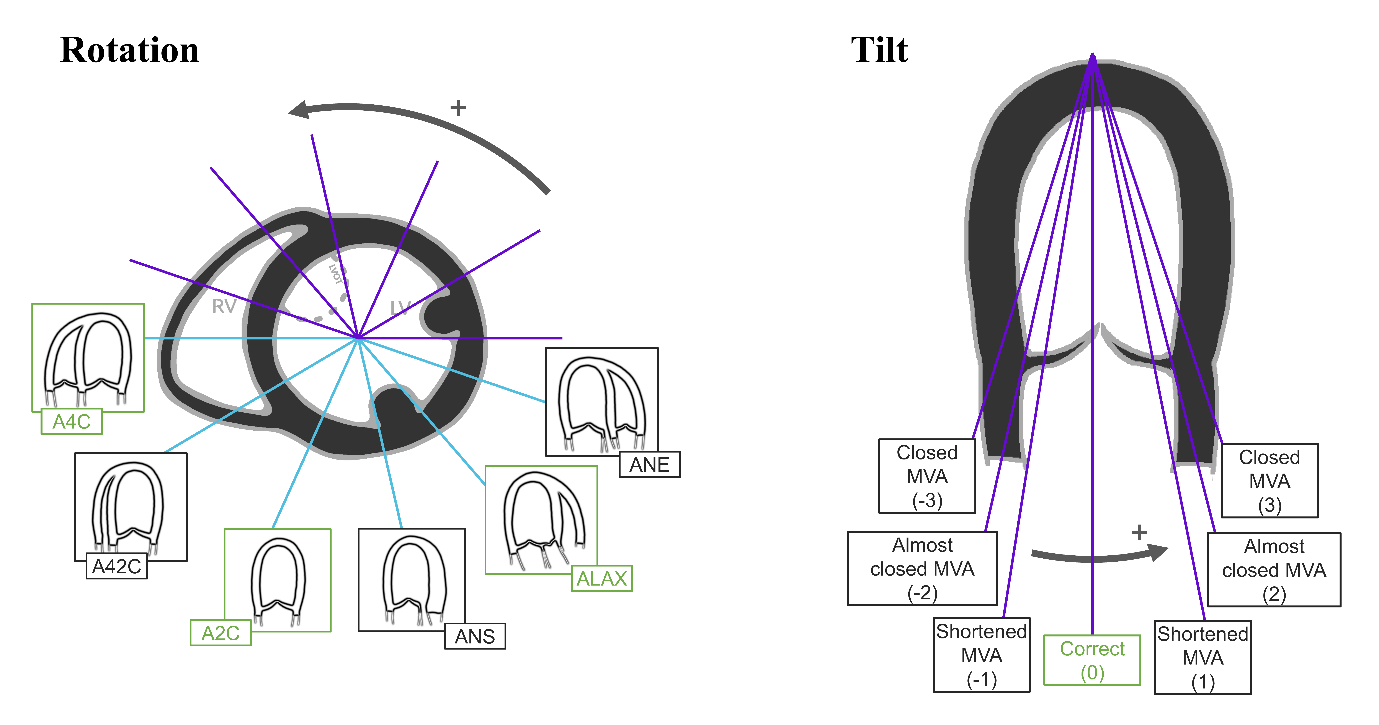


The scoring corresponds with the contents of Supplemental Table 1. The arrow marked with the plus sign indicates the direction of the scoring scale. Abbreviations: A4C, Apical four-chamber; A2C, Apical two-chamber; A42C, rotational position where the right ventricle appears/disappears; ALAX, Apical long axis; ANE, Aortic valve annulus end; ANS, Aortic valve annulus start; MVA, Mitral valve annulus.

**Supplemental Figure 2.** Operator-specific distributions for A2C rotation. **
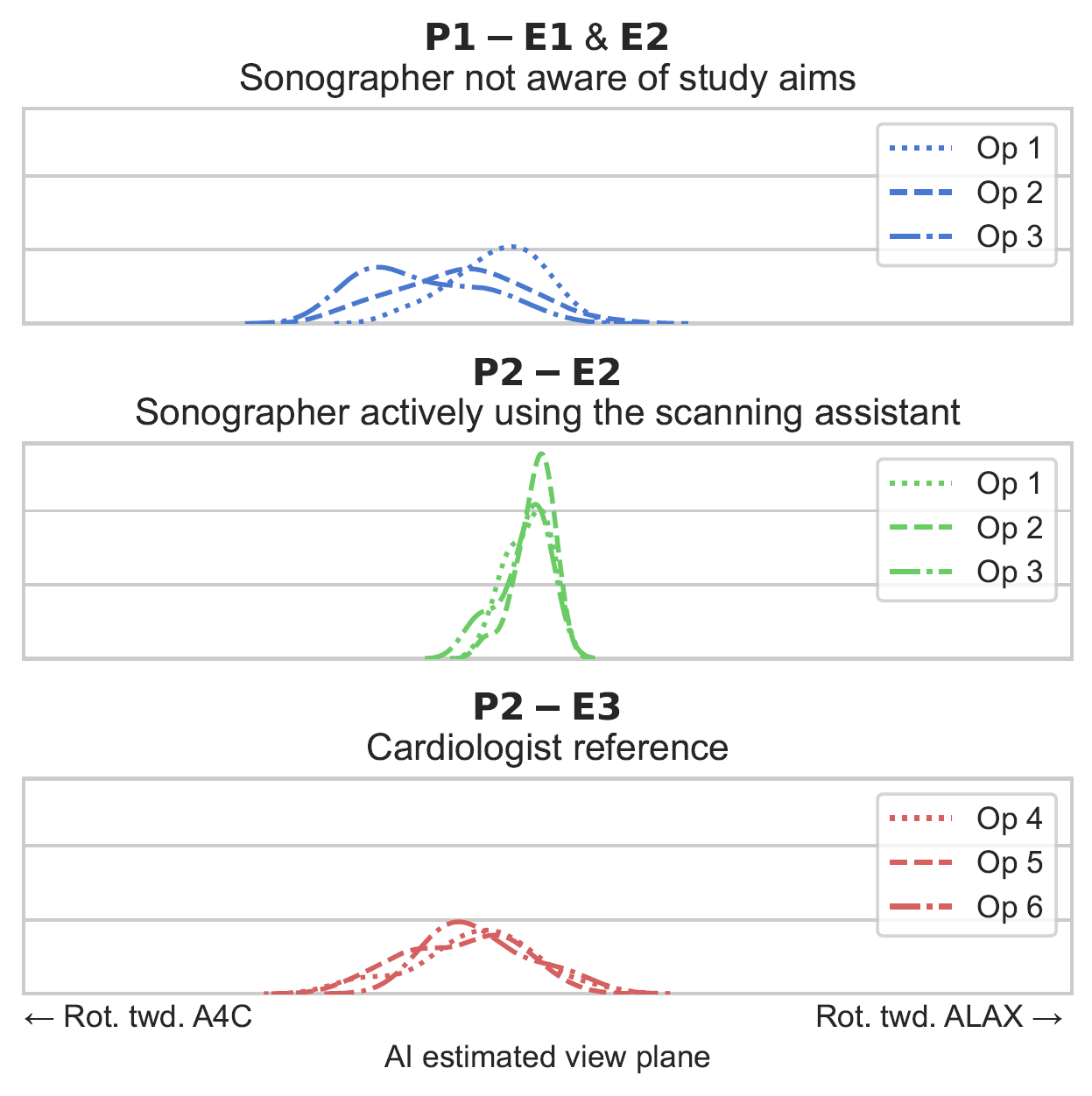
**

Abbreviations: A2C, Apical two-chamber view; A4C, Apical four-chamber view; ALAX, Apical long axis view; E1, Examination 1; E2, Examination 2; Op, Operator; P1, Study Period 1; P2, Study Period 2; Rot. twd, Rotation towards.

**Supplemental Figure 3.** Operator-specific distributions for A2C tilt.
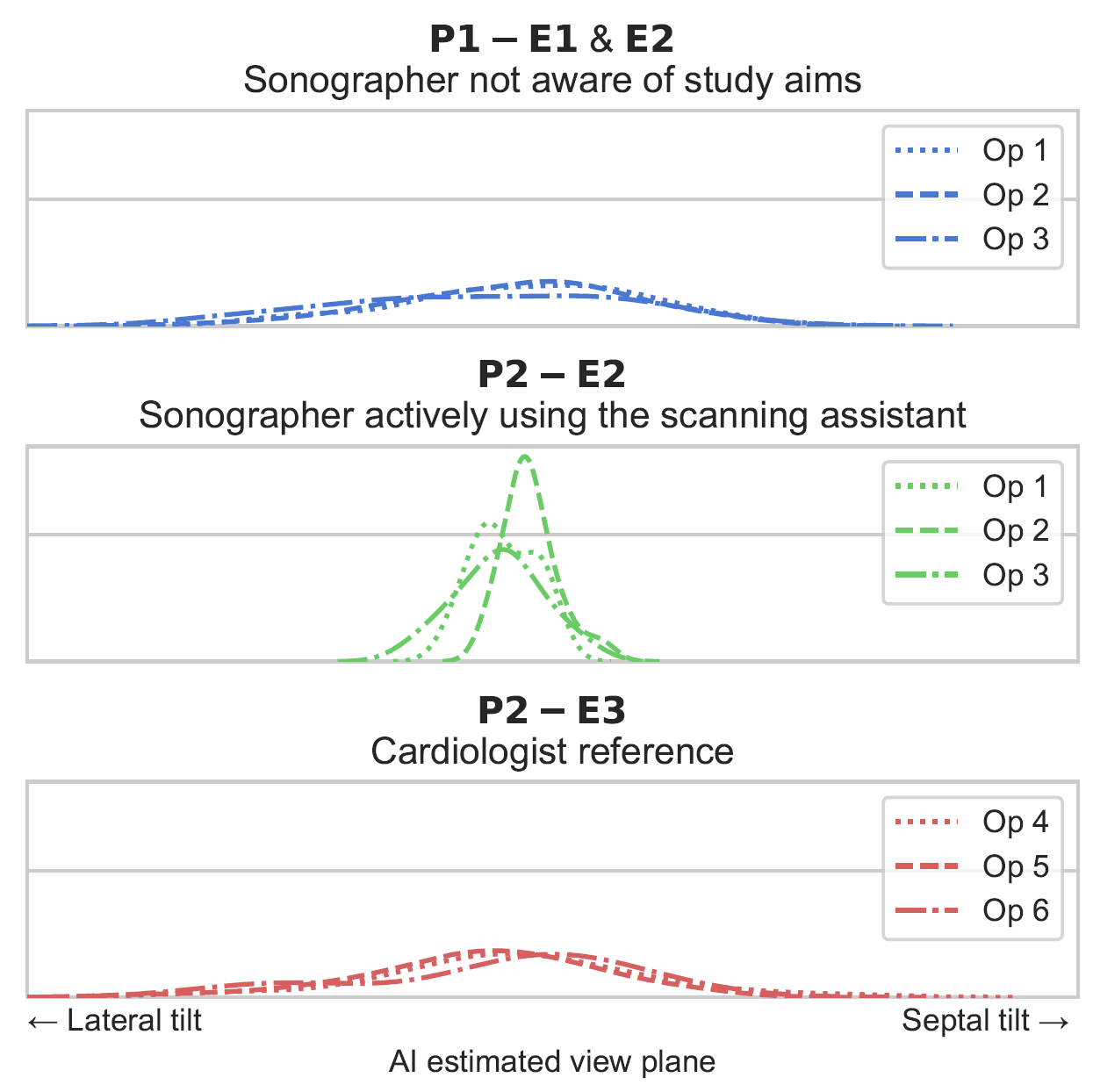


Abbreviations: A2C, Apical two-chamber view; E, examination; Op, Operator; P, Study Period.

**Supplemental Figure 4.** Operator-specific distributions for A4C rotation.
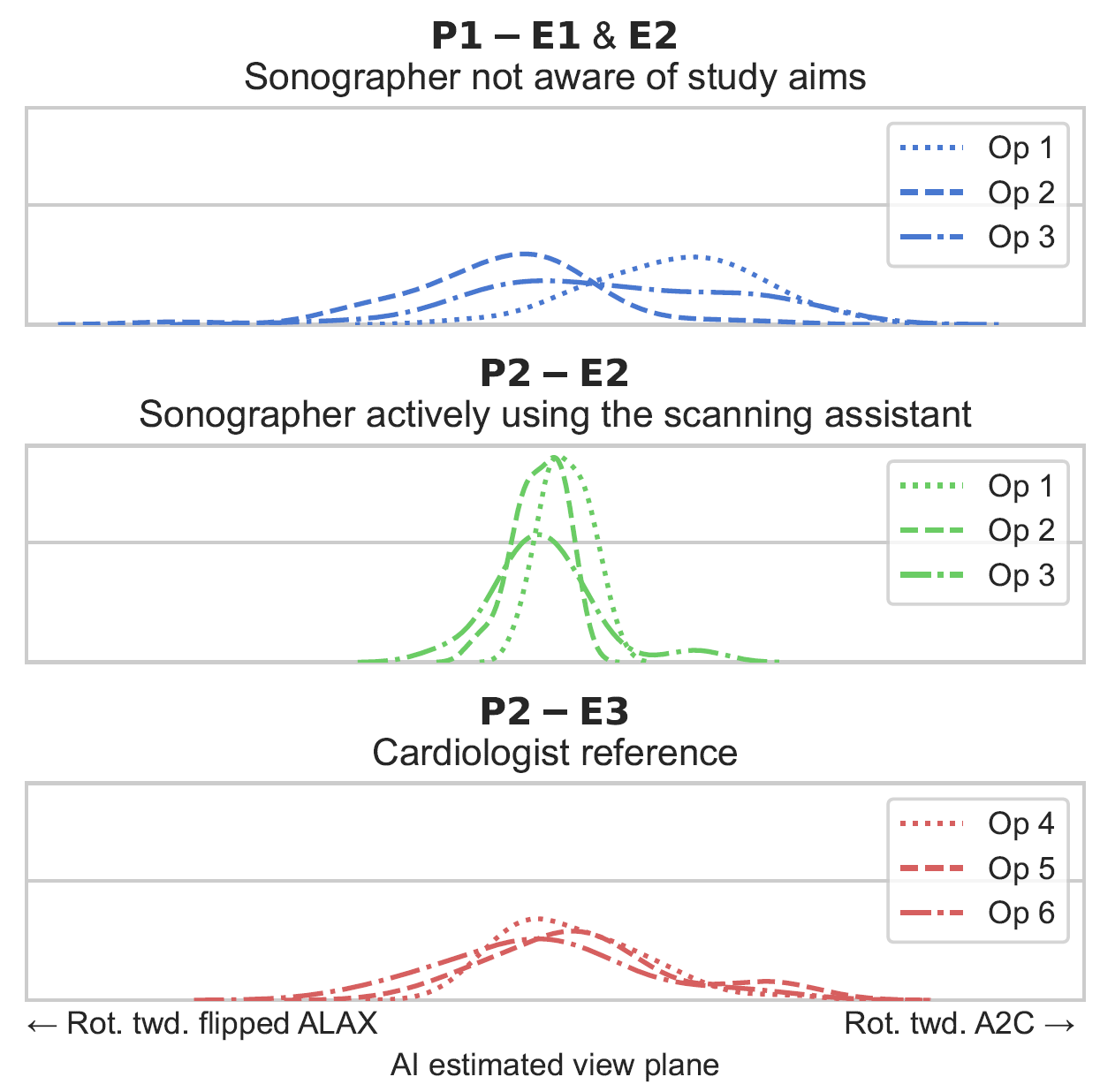


Abbreviations: A2C, Apical two-chamber view; A4C, Apical four-chamber view; ALAX; Apical long axis view E, examination; Op, Operator; P, Study Period; Rot. twd, Rotation towards.

**Supplemental Figure 5.** Operator-specific distributions for A4C tilt.
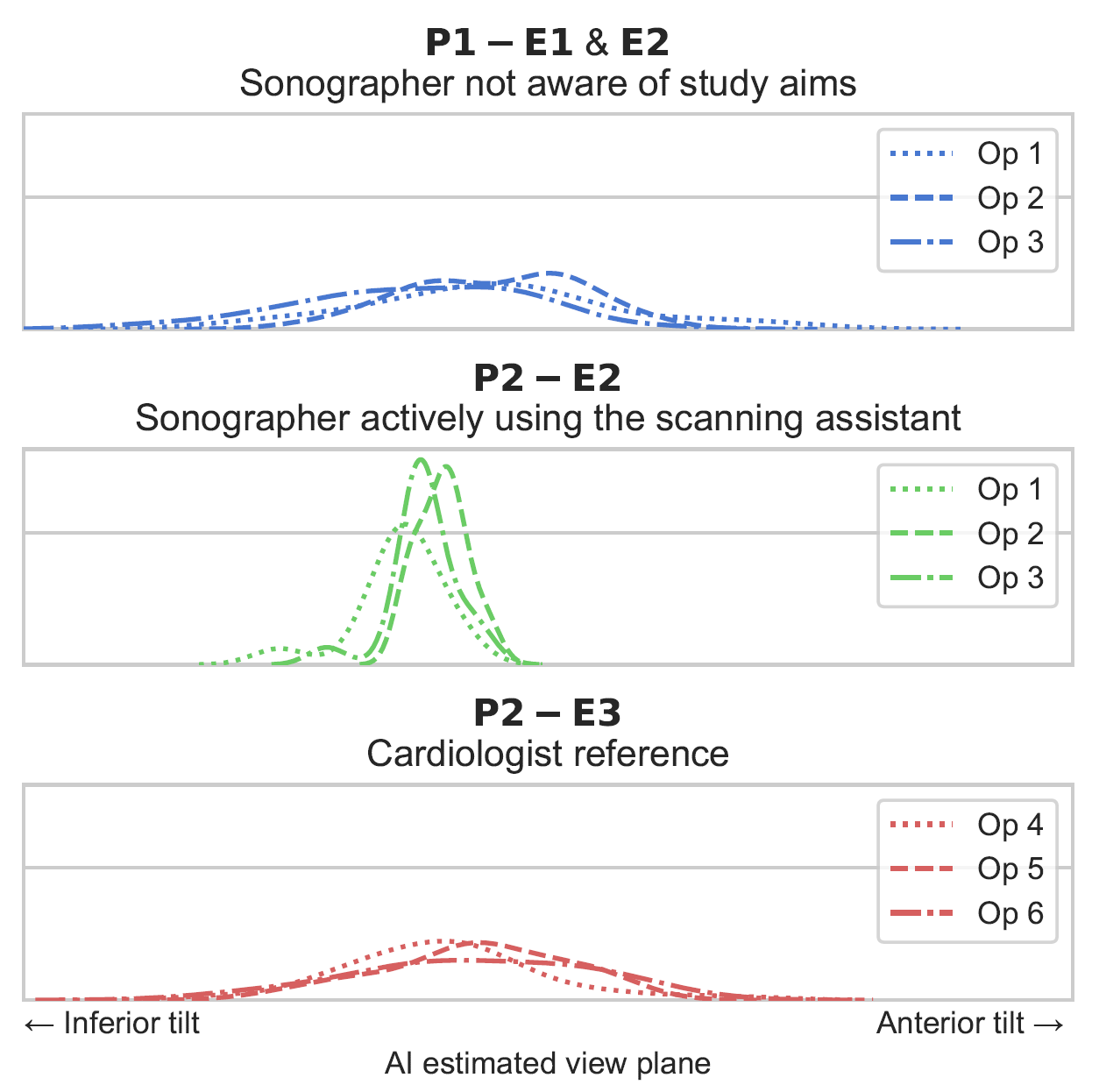


Abbreviations: A4C, Apical four-chamber view; E, examination; Op, Operator; P, Study Period.

**Supplemental Figure 6.** Operator-specific distributions for ALAX tilt.
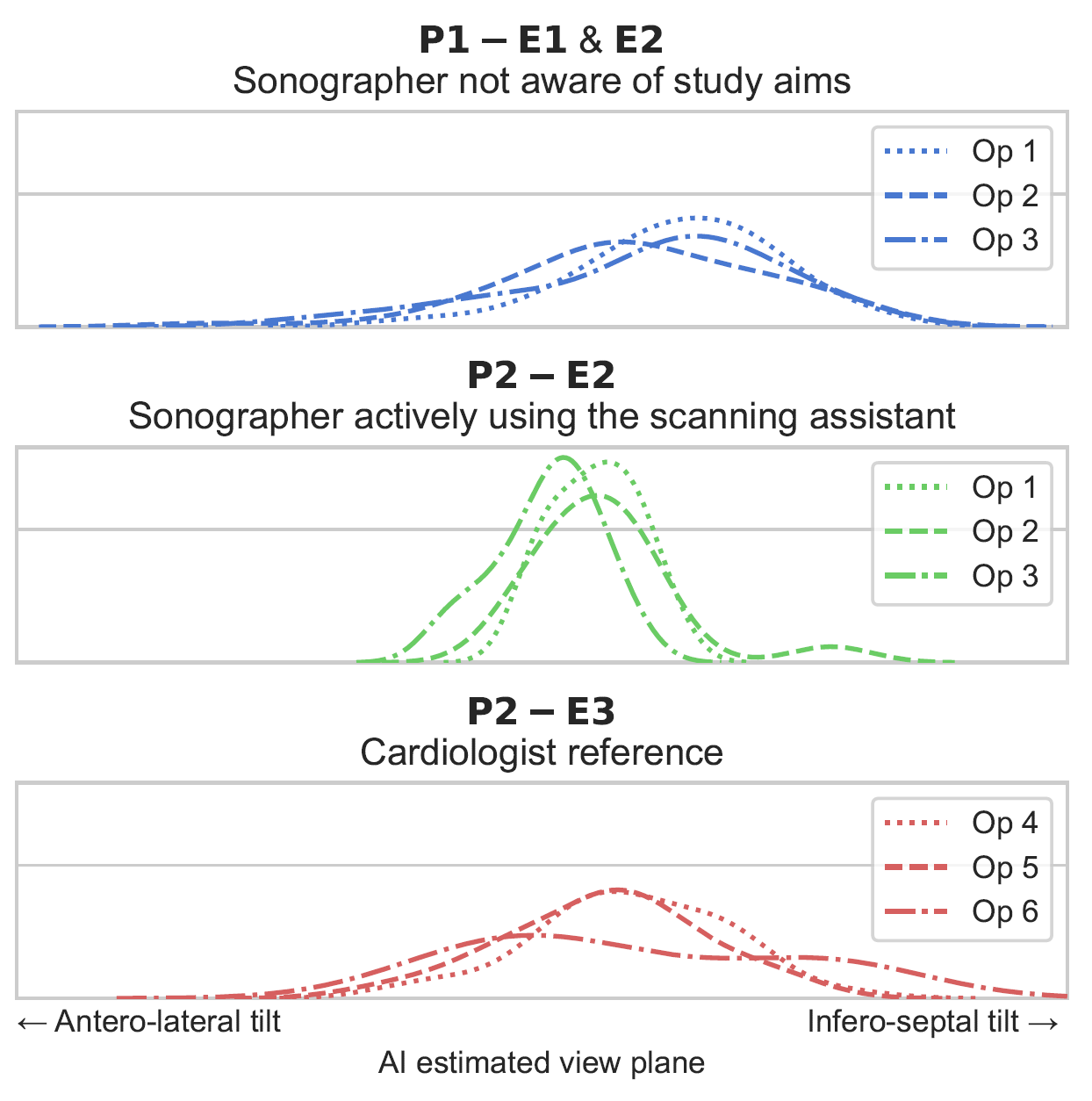


Abbreviations: ALAX, Apical long axis view; E, examination; Op, Operator; P, Study Period.

**Supplemental Figure 7.** Proportion of examinations by different sonographers according to study period and examination order.


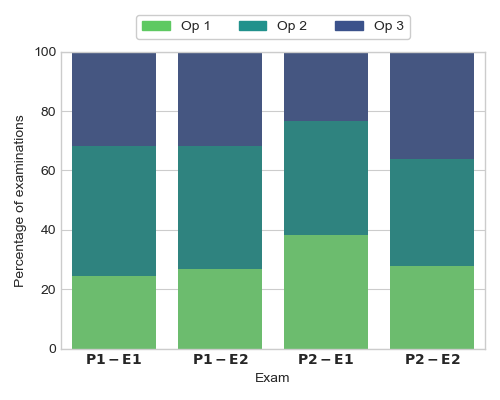


Abbreviations: Op, Operator; P, Study Period

**Supplemental Table 1.** Scoring system for the human expert evaluation of standardization.

| View | Grading rotation | Grading tilt |
| --- | --- | --- |
| Apical four-chamber | 3= >A42C  2 = A42C  1 = <A42C  0 = A4C  -1= <-ANE  -2 = -ANE  -3 = >-ANE | 3 = apical five-chamber 2 = more anteriorly, aortic valve visible  1 = anteriorly, LVOT visible  0 = correct -1 = shortened mitral annulus  -2 = almost closed mitral annulus -3 = closed mitral annulus |
| Apical two-chamber | 3 = >ANS  2 = ANS  1 = <ANS  0 = A2C  -1 = <A42C  -2 = A42C  -3 = >A42C | 3 = closed MVA+ open AV  2 = almost closed MVA  1 = shortened MVA/LVOT  0 = correct  -1 = lateral, some anterior PM  -2 = more lateral, more PM  -3 = closed MVA |
| Apical long axis | 3 = >ANE  2 = ANE  1 = <ANE  0 = correct  -1 = <ANS  -2 = ANS  -3= >ANS | 3 = closed MVA  2 = almost closed MVA  1 = shortened MVA  0 = correct  -1 = some anterolateral, shortened AV  -2 = more anterolateral, no AV  -3 = closed MVA, no AV |

The scores’ direction and deviation from 0 corresponds with the illustration in Supplemental Figure 1. The integers are landmarks, but the scale was continuous. The rotational and tilt direction and landmarks are illustrated in Supplemental Figure 1. Abbreviations: A4C, Apical four-chamber; A2C, Apical two-chamber; A42C, rotational position where the right ventricle appears/disappears; ALAX, Apical long axis; ANE, Aortic valve annulus end; ANS, Aortic valve annulus start; AV, Aortic valve; LVOT, Left ventricular outflow tract; MVA; Mitral valve annulus; PM, Papillary muscle; RV, Right ventricle.
