# Supplementary figures and images for: Real-time guidance by deep learning of experienced operators to improve the standardization of echocardiographic acquisitions
Source: Eur Heart J Imaging Methods Pract. 2023 Nov 27;1(2):qyad040. doi: 10.1093/ehjimp/qyad040 (PMC11195719; doi:10.1093/ehjimp/qyad040)

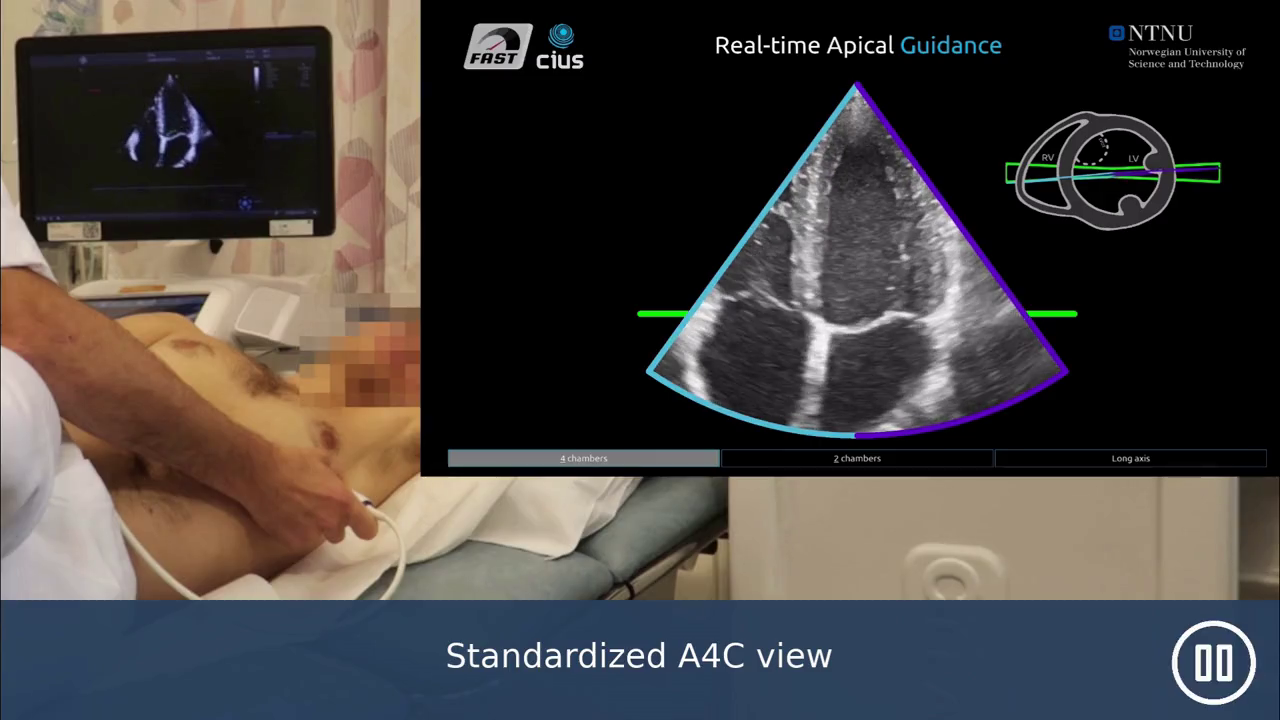

Supplement: qyad040_Supplementary_Data [file qyad040_Supplementary_Data.zip › video1_snapshot.png]

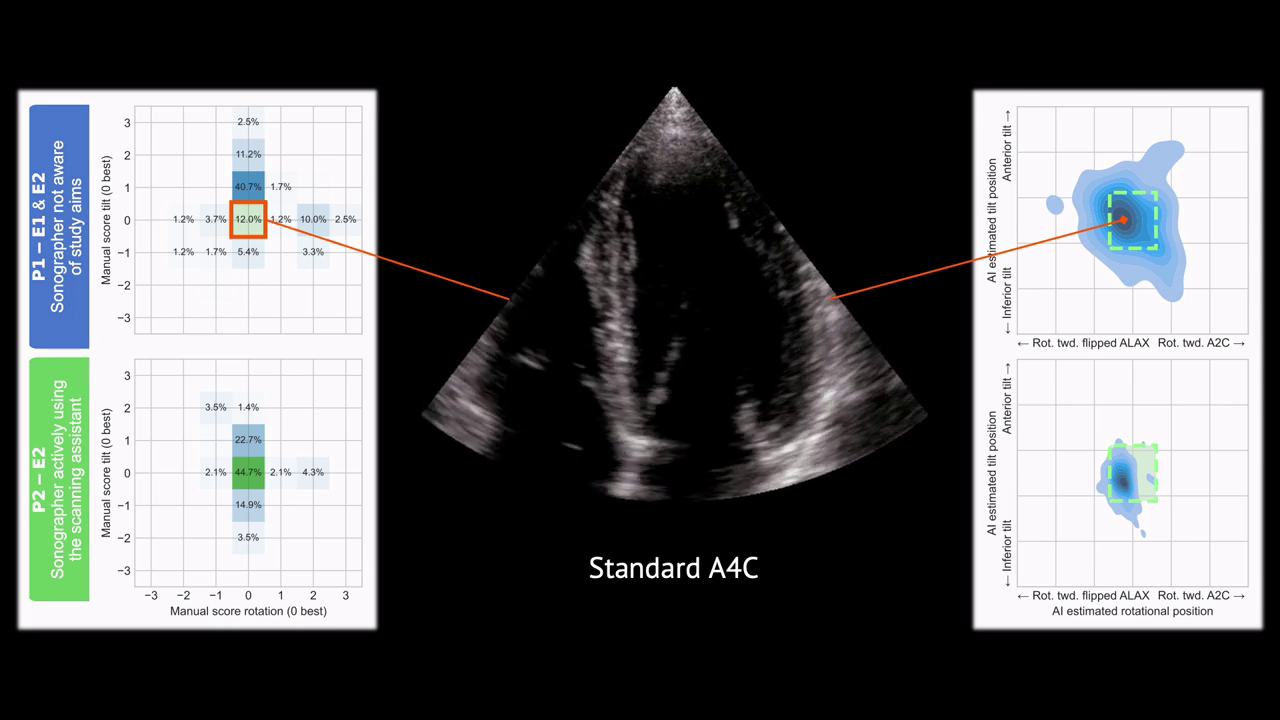

Supplement: qyad040_Supplementary_Data [file qyad040_Supplementary_Data.zip › video2_snapshot.png]
